# Management research and the impact of COVID-19 on performance: a bibliometric review and suggestions for future research

**DOI:** 10.1186/s43093-022-00149-1

**Published:** 2022-09-30

**Authors:** Kingsley Opoku Appiah, Bismark Addai, Wesley Ekuban, Suzzie Owiredua Aidoo, Joseph Amankwah-Amoah

**Affiliations:** 1 School of Business, Kwame Nkrumah University of Science and Technology, Kumasi, Ghana; 2 School of Economics and Management, Changsha University of Science and Technology, Changsha, China; 3 Department of Accounting, FSI Chartered Accountants and Advisory, Accra, Ghana; 4 Department of Public Leadership and Social Enterprise (PuLSE), The Open University, Milton Keynes, UK; 5 Kent Business School, University of Kent, Kent, UK

**Keywords:** Pandemic, Management research, COVID-19, Bibliometrics, Epidemic, Coronavirus

## Abstract

Although there has been a burgeoning scholarly interest in the effects of COVID-19, the current stream of research remains scattered in different business and management fields and domains. Accordingly, integrative knowledge is needed to drive poignant and relevant examinations of the phenomenon. This study attempts to fill this gap by providing a synthesis of the literature, patterns of research studies, and direction for further development of the field. This study also provides a systematic identification and bibliometric and thematic review of literature, performance analysis, science mapping, and cluster analysis. The study additionally provides suggestions for future research to guide relevant discourse.

## Introduction

The term pandemic has been used to describe the widespread outbreak of disease through human-to-human infections [1]. Medical texts providing a clear definition of what constitutes a pandemic are non-existent. However, its geographic extent and infectiousness and severe negative impact on all aspects of society are clearly understood [2]. To this end, research has continued to understand the extent to which pandemics shape communities, economies and society as a whole [3, 4]. The COVID-19 pandemic is no exception, with its catastrophic effects considered to be one of the worst in human history [5]. It is no surprise the plethora of studies seeking to understand the phenomena. The severity of the pandemic has paved the way for a rapidly escalating body of empirical literature analyzing the consequences of the COVID-19 pandemic on countries [6, 7], firms [8], and households [9, 10]. Some business and finance-related studies, for example, have focused on macro-economic indicators [11], policy alternatives and implementation [12, 13], business responses and implications [4, 14] as well as firm performance outcomes and failures [15, 16].

The COVID-19 pandemic like all other global crises impacts on all aspects of life including business activities. Shocks caused by such events disrupt business operations across the globe and in extreme situations lead to business failure [17, 18]. The influence of business activity on national and global economies has encouraged an increasing scholarly interest in understanding the extent to which firm performance has been impacted. Developing suitable and sustainable policy and strategy responses is the logical action and focus of all governments and scholars to help mitigate the negative effects of this pandemic. However, to develop such effective strategies, the extent and various ways in which firm activities and performance has been affected must first be examined. To this end, scholars have found that the COVID-19 pandemic has severely impacted the performance of the hospitality industry [19], supply chains [20], stocks of listed companies [21, 22], SMEs and family firms [23, 24].

Notwithstanding the vital contributions of these studies, the integral role of research is to detect and synthesize patterns, conditions and effects in business activity, to help ensure effective decision-making and policy development. Carracedo et al. [25] began this pattern detection by conducting a systematic literature review of relevant literature. Although, Carracedo et al.’s [25] study offers novel insights into the clusters of COVID-19 business-related studies, it hardly provides in-depth knowledge and practicable knowledge on the scope, relationships and gaps in literature. To this end, the present study advances knowledge by conducting a systematic literature review and bibliometric analysis of the relationship between COVID-19 and firm performance.

The contribution to the literature is threefold. First, based on the review a comprehensive baseline systematization on the impact of COVID -19 on firm performance was advanced to enhance the understanding of the impact of the pandemic on firm performance. In so doing, we also provide fruitful lines for future research and/or policy (see [26]). Second, synthesizing the rapidly evolving literature into a conceptual framework/clusters, the study provides academicians, industrial players, government agencies, and all other stakeholders a comprehensive overview and access to the central topics, trends and the implications of the research on the impact of the pandemic on firm performance. Furthermore, the review of the data provides an opportunity to offer a deeper insight to help control the impact of the pandemic on firm’s performance and the antecedent effects on households and economies.

The rest of the paper proceeds as follows. “Method and initial statistics” section discusses the method. “Bibliometric analysis” and “Thematic/cluster analysis” sections present an in-depth bibliometric and cluster/thematic analyses of the dataset, respectively. “Directions for future research” and “Conclusion and limitations” sections provide direction for future research and the conclusion, respectively.

## Method and initial statistics

The objective of this study is to construct a scientific map and further analyze the worth of knowledge produced by management experts who examine the impact of COVID-19 on firms’ performance. Following relevant literature (e.g., [27, 28]) and best practices in the mapping of scientific knowledge, we conduct a bibliometrix analysis and a systematic review of the relevant literature. Specifically, we use the bibliometrix to construct scientific mapping to highlight the knowledge base, and its intellectual structure as well as both the conceptual and social network structures of Covid 19’s impact on performance extant literature (see [29, 30]). By combining the two complementary approaches, we are able to paint a picture of the development of scientific knowledge on the impact of COVID-19 on firm performance using quantitative bibliometrix tools and also provide a comprehensive analysis of the themes/topics and contents by means of qualitative systematic review. These approaches are well established in management literature (see [27, 31]).

### Data collection

To undertake this systematic and bibliometric analysis, articles discussing the influence of COVID-19 on firm performance were retrieved and analyzed. To ensure and maintain an unbiased and high-quality database and review, strict criteria were adhered to. These are described in the following steps:

- **Step 1** A literature search was conducted in Scopus Database. The aim is to ensure broader access to ranked management related, reputable and quality journals.
- **Step 2** The search was conducted with the search term: ("COVID-19" or "CoronaVirus") and ("value" or "performance" or “profitability”). Various search strings using multiple combinations of the search terms were used during the data collection process. These include ((covid-19 OR coronavirus) AND (value OR performance OR profitability)).
- **Step 3** The search focused on scholarly studies relating to the impact of COVID 19 on firms published from 2019 to 24 July, 2021. We consider papers published from 2019 to 2021 because COVID-19 emerged as a global health crisis in 2019 [9] and we finished our literature search in July 2021.
- **Step 4** These studies were limited to final articles published within Business & Management & Accounting, Social Science, Economics, Econometrics & Finance Journals. We omit books, Ph.D. Thesis, working papers, technical reports, conference proceedings. The stages and the tasks undertaken during the literature search are summarized in Table 1.

**Table 1:** Literature search and inclusion results

Stage	Task	Articles
1	Literature search in Scopus	19,645
2	Limited to 2019–2021	18,710
3	Limit to business management and accounting	1571
4	Limit to published articles	1372
5	Limit to English articles	1355

An initial search produced 19,645 articles, excluding articles outside of the 2019–2021 range yielded 18,710. Of these 1571 were management and accounting related. Subsequently, review papers (70), conference papers (59), editorial (10), book chapters (14), note (27), book (7) and letters (4) leaving 1372 articles. Finally, 1355 English journal articles were retained for the bibliometric and thematic analyses. Subsequent steps in this study consisted of conducting a bibliometric and a thematic analysis of articles retained. The bibliometric analysis consists of a performance analysis of articles, authors and journals to identify relevant literature in the field. Next a scientific mapping of country production as well as a keyword analysis is conducted to highlight on the various research topics in the field. We use Biblioshiny and VOSViewer Software applications to perform the bibliometric analysis. Finally, the study conducts a detailed thematic analysis, by identifying and synthesizing studies within the four main research clusters.

### Descriptive statistics

Table 2 captures the description of the data collected for the bibliometric analysis and the SLR. As illustrated in the table, we identify 1355 scholarly articles, spread across 437 sources, with 73,478 references, 4407 authors keywords, 3538 authors, 0.383 article per author, and 2.85 co-authors per article. Our descriptive also shows 253 single authorships, while collaboration index is 3.03 (see Table 2). Our analysis shows the impact of Covid 19 on performance was first mentioned in the Management research literature in 2019 by Sterling and Merluzzi’s (2019) paper, highlighting that tryouts may rise due to Covid 19-related impacts on US Firms. Table 2 reports an astronomical rate of 31.7% for the 2-year period from 2020 to 2021, implying the ever-increasing literature on the impact of Covid 19 on performance.

**Table 2:** Main information of the data collected as at 24/07/2021. *Source*: R-tool for Bibliometrix: Biblioshiny, 2021

Description	Results
Article	1355
Period	2019–2021
Annual percentage growth rate	31.7
Average citations per article	3.517
Authors	3535
Author appearances	3862
Authors of single-authored articles	253
Authors of multi-authored articles	3282
Authors per article	2.61
Articles per author	0.383
Co-Authors per articles	2.85
Collaboration index	3.03
Sources	437
Average citations per year per article	2.178
References	73,478
Keywords plus (ID)	1189
Author's keywords (DE)	4407

The study examines the scientometric index measuring a journal’s impact by assessing the average number of article citations over the last two years. Table 3 shows the top ten most impactful journals in terms of the number of publications on impact of Covid-19 on performance research. It should be noted that the top ten journals published 215 out of 1355 articles, accounting for 15.9% of articles in our dataset. The results indicate that majority of these journals ranked three or two in Association of Business School Journal quality list. Overall, these multi-disciplinary outlets suggest the topic is attractive to all management scholars and research areas.

**Table 3:** Top 10 journals in terms of number of publications on Covid 19 and performance

Journal	Articles	%
International Journal of Contemporary Hospitality Management	35	15.22
Journal of Public Budgeting Accounting and Financial Management	31	13.48
Accounting Auditing and Accountability Journal	29	12.61
Journal of Asian Finance Economics and Business	21	9.13
Worldwide Hospitality and Tourism Themes	21	9.13
Accounting Research Journal	17	7.39
Journal of Accounting and Organizational Change	16	6.96
International Journal of Hospitality Management	15	6.52
Journal of Service Management	15	6.52
Journal of Tourism Futures	15	6.52
Tourism Review	15	6.52

## Bibliometric analysis

The study’s bibliometric analysis reveals the valuable insights to knowledge within management research in assessing the influences of COVID on business performance.

### Author influence

This section discusses the most impactful authors to the research domain. Table 4 displays the most impactful author on the basis of h-index, m-index, g-index and number of publications. We examine author’s impact by analyzing the number of academic benchmark performance indicators, namely citations, H-index, G-index, and M-index [32–34]. The H-index, viewed as an unbiased overview, for example, combines the number of papers and citations to assess author’s scientific contributions over time [35]. The H-index, however, overlooks the impactful but discriminatory author, implying it favors high-volume authors. Accordingly, we complement the H-index analysis with the G-index. G-index measures the highest rank such that the top G papers have, together, at least G^2^ citations (see [35] for details).

**Table 4:** Top 10 impactful authors on Covid-19 and performance

Author	H	G	M	TC	NP
Dmitry Ivanov	5	6	2.5	448	6
Vanessa Ratten	4	8	2	64	8
J. Andres Coca-Stefaniak	3	3	1.5	23	3
Sertan Kabadayi	3	3	1.5	45	3
Jungkeun Kim	3	3	3	11	4
Alastair M Morrison	3	3	1.5	23	3
Kun Yang	3	3	1.5	28	3
Herman Aguinis	2	2	1	19	2
Joseph Amankwah-Amoah	2	2	1	9	2
Tom Baum	2	2	1	116	2

TC, NP, H, G, and M denote total citation, number of publications, H-index, G-index and M-index

Overall, the small number of publications from these prolific authors confirms the infancy stage of the research domain, thereby allowing different authors with diverse expertise in management research to contribute to the discourse on the impact of Covid-19 and performance from 2019 to present. The results indicate that *Dmitry Ivanov* is the most influential author with an H-index of 5 and a G-index of 6. All 6 articles authored or co-authored by him have received a total of 448 citations. This is followed by *Vanessa Ratten* with H and G-indexes of 5 and 6, respectively. She has authored and co-authored a total of 8 articles in the field, earning a total citation score of 64. Next, *Andres Coca-Stefaniak, Sertan Kabadayi and Jungkeun Kim* assume the third, fourth and fifth most impactful author, respectively, each with an H and G-index of 3. Generally, the H and G-indexes produce different rankings, the results show that the ranking remains the same except for the highest and second highest ranked authors, who interchange according to the index understudy.

Again, critics argue that both H-index and G-index ignore different career lengths [36]. Accordingly, we use the M-index (i.e., M-quotient), which is H-index divided by author’s active literary years to help provide a clearer view of author rankings in the research area. Ranking scholars according to the M-quotient, sees *Junkeun Kim* as the highest ranked author with an M-quotient/index of 3, *Dmitry Ivanov* and *Vanessa Ratten* are the second and third highest ranked authors. Other impactful authors, who have contributed immensely to the field and are ranked among the top 10 impactful scholars are summarized in Table 4.

### Geographical and institutional scientific production

Biblioshiny was used to retrieve the author organization/affiliation and address information, after which all the authors were sorted in descending order. Table 5 displays the top 20 organizations publishing the most articles on Covid 19 and performance. University of Johannesburg in *South Africa* contributes most with 17 articles on Covid 19 and performance, followed by *The Hong Kong Polytechnic University* in *Hong Kong*. Table 4 shows that RATTEN V from the La Trobe University contributes most with 9 articles, and the affiliation appears as the Top 6 contributing organization. Surprisingly, most of the other influential authors do not have their organizations listed in Table 5. For example, *Ivanov D, Gupta S, Kim J, Kumar A, Li S, Li Z, Sharma A, Zhang J,* emerge as the Top 2 to 5 contributing authors, respectively; however, their organizations do not appear in influential organizations in Table 5. Likewise, the University of Johannesburg emerges as the Top 1 contributing institution with 17 publications but no author from this university is listed in Table 4. Similar surprises exist at other universities such as the *RMIT university*, the University of Auckland and the Auckland University of Technology which appear in Table 5 but no author from those organizations appears in Table 4. The results imply that the contributing authors have come diverse research backgrounds in different methodological and industrial settings. For instance, the 17 publications from the *University of Johannesburg* cover various industries such as fruit, food, manufacturing and food, and they employ differing methodologies including empirical analysis and case studies.

**Table 5:** Top 20 organizations contributing to the topic of Covid-19 and Performance

Organizations/institutions	Number of publications
University of Johannesburg	17
The Hong Kong Polytechnic University	16
Rmit University	15
The University of Auckland	13
Auckland University of Technology	12
La Trobe University	12
Queensland University of Technology	10
Universitas Indonesia	9
University of Central Florida	9
Victoria University of Wellington	9
Copenhagen Business School	8
Macquarie University	8
Universiti Sains Malaysia	8
University of The Punjab	8
Edith Cowan University	7
Griffith University	7
Sejong University	7
The University of Melbourne	7
Universitas Gadjah Mada	7
University of Bologna	7

**Fig. 1:**
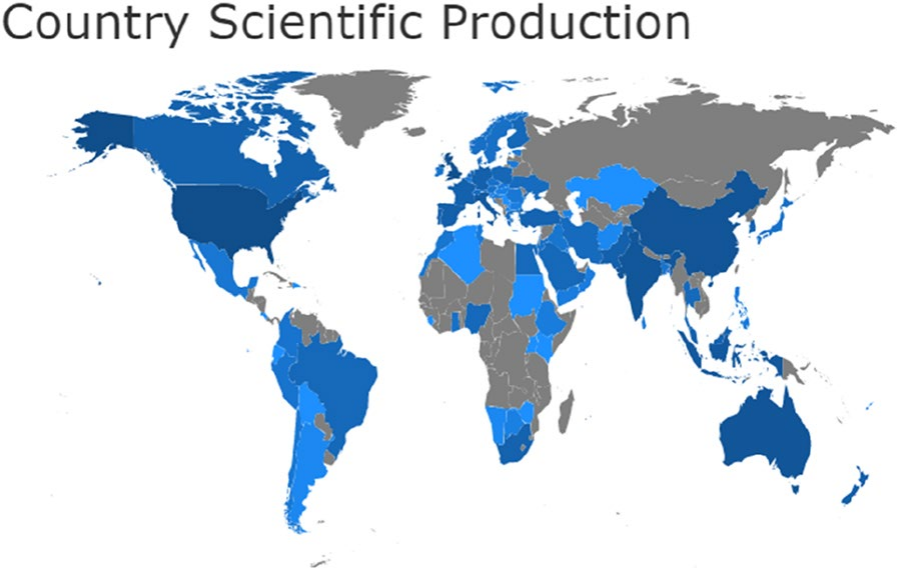
Geographic dispersion of publications on Covid-19 and performance

Being a global health issue that has induced economic and social upheaval worldwide, COVID-19 and its related performance impact has received global scholarly attention. The geographic distribution of research attention is shown in Fig. 1. The United States of America has the highest concentration of COVID-19 and performance impact studies of 377 (22.93%) articles. The literature on performance impact of COVID-19 has received much attention in the United States because the country has encountered disproportionately elevated levels of economic fallout, infections and deaths [6, 37]. For instance, WHO reports that compared to other countries, the USA has the highest infection and death rates of 99,085,620, and 2,377,656, respectively. The United Kingdom emerges as the second country with concentration of studies on the performance impact of COVID-19 contributing 286 (17.40%) of the articles. China, Australia and India contribute the third (11.50%), fourth (10.95%) and fifth (10.10%) positions, respectively. Also, the impact of Covid-19 is evaluated in the Indonesia, Italy, New Zealand, Spain and Portugal by other 446 empirical research articles, representing 27.14% of the top 10 geographic dispersion. Generally, the geographical distribution in Fig. 1 depicts that countries in North America (USA), Oceania, Europe and Asia have the highest interest in studies on the performance impact of COVID-19 as compared to those in South America, Africa and Antarctica (where the interest in the study area is scanty). However, literature shows the highly interrelated nature of the global economy today [38]. Besides, the multinational nature of business activities today, heightens the need to expand knowledge on performance impacts across all continents. Thus, more empirical analysis on the impact of Covid-19 on performance is needed in those regions seemingly underrepresented in scholarship but pivotal to global business and supply chain [39, 40].

## Keyword analysis

Next, we conduct a keyword analysis to identify popular research perspectives in the area. Table 6 contains the biblioshiny’s keyword analysis results of 4407 author keywords in the 1355 articles reviewed in the study. As expected, COVID-19 and several iterations of the *virus, crisis, crisis management* and *resilience* make up the top 8 keywords. “*Tourism”* takes 9th place on the list, indicating the relevance of tourism to national and global economies and elucidating the extent to which the pandemic has influenced the valuable global industry [41]. Innovation and resilience have been frequently discussed in conjunction with COVID-19 and business research, highlighting inherent relations between innovation and resilience in the COVID-19-performance nexus [42, 43]. Again, Finsterwalder and Kuppelwieser [44] and Golan et al. [39] highlight the need for resilience and for developing appropriate strategy for recovery following a disruptive event. “Crisis Management” and “Leadership” are similarly popular research foci owing to the significant roles crisis management and leadership play in mitigating crisis and lessening its adverse effects [45, 46]. Other frequently used keywords include performance, entrepreneurship, stock market, and supply chain as they all relate to business management and the performance implications of the pandemic. Country-related keywords include India and China, which have both been epicenters of the pandemic at one point or another [47]. Lastly, Table 6 shows gender as a frequently used keyword as a result of the recorded differences in infection and fatality rate among men and women [48] as well as employment, equality and other sociological equality implications [49].

**Table 6:** Most frequently used keywords in the topic Covid-19 and Performance

Keywords	Frequency	Keywords	Frequency
Covid-19	695	Performance	20
Pandemic	103	Entrepreneurship	18
Covid-19 Pandemic	78	Innovation	18
Coronavirus	74	Lockdown	18
Crisis	51	Stock Market	17
Resilience	47	Supply Chain	17
Crisis Management	37	Gender	16
Sustainability	33	India	16
Tourism	27	Social Media	16
Leadership	20	China	15

**Fig. 2:**
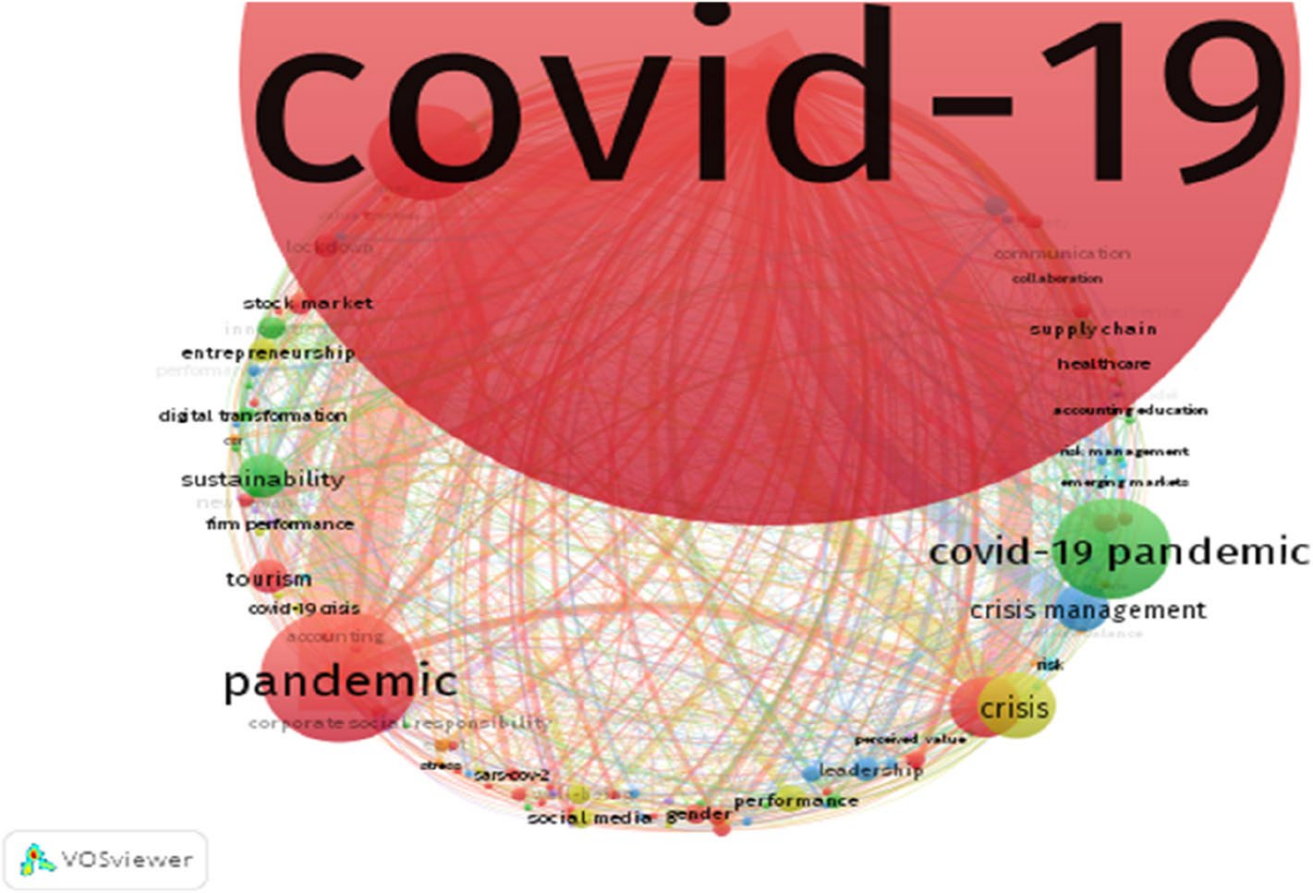
Network visualization diagram of authors keyword on Covid-19 and performance

To further identify themes investigated in the management research and the impact of COVID -19 and Performance, given that the field is at its infancy, we used co-occurrence of keywords analysis based on keywords that occurred at least five times, resulting in 133 satisfying the threshold out of 4407 authors keywords. Figure 2 displays the network visualization diagram of VOSviewer highlighting seven common keywords, namely, COVID-19, pandemic, coronavirus, resilience, crisis, COVID-19 pandemic and crisis management. The size of nodes and thickness of line displayed in Fig. 2 verify these findings (see [50], p. 552 for further reading). The homogeneous nature of these keywords confirms the collective focus on business performance and economic impacts of the pandemic. Figure 2 also displays 7 clustered keywords for 132.

### Citation analysis

In analyzing the 1355 articles, the study examined the citation measure of each article. Citation analysis is usually measured using two indexes: the global and local citations, whereas the former represents the citation of a given article by other articles within the entire academic database of articles, the latter indicates the citation score of a given article by other articles within the articles being assessed in this study [29].

Table 7 reports the top 10 cited articles, based on both local and global citation scores. The table shows that the most impactful article is *“Predicting the impacts of epidemic outbreaks on global supply chains: a simulation-based analysis of the COVID-19/SARS-CoV2 case"*—[51]*.* Ivanov [51] examines the global supply chain impacts of pandemic outbreak through simulation-based analysis, which was published in Transportation Research Part E: Logistics and Transportation Review. The study focuses on crucial management and performance issues affected during crisis such as risk management and resilience and thus sets the tone for the later empirical studies. The second most cited article is “*Risk perceptions of COVID-19 around the world*”—[52]. This article mapped and modeled the risk perception of COVID-19 around 10 countries (US, UK, Australia, Germany, Spain, Italy, Japan, South Korea, Mexico, and Sweden). It highlights the role strong predictive roles of various experiential, and socio-cultural values and factors. The next highest cited article is “*Tourism and COVID-19: Impacts and implications for advancing and resetting industry and research*”—[53]. The study critically evaluates tourism transformation and impacts of the pandemic through a literature review and sets the tone for resetting and advancing research frontiers.

**Table 7:** Top 10 cited articles of Covid-19 and Performance

	Article	LC	GC
1	Predicting the impacts of epidemic outbreaks on global supply chains: A simulation-based analysis on the coronavirus outbreak (COVID-19/SARS-CoV-2) case	326	163
2	Risk perceptions of COVID-19 around the world	256	128
3	Tourism and COVID-19: Impacts and implications for advancing and resetting industry and research	179	89
4	Feverish stock price reactions to COVID-19	89	44
5	Effects of COVID-19 on hotel marketing and management: a perspective article	88	44
6	COVID-19: potential effects on Chinese citizens' lifestyle and travel	82	82
7	The economics of COVID-19: initial empirical evidence on how family firms in five European countries cope with the corona crisis	82	41
8	Employee adjustment and well-being in the era of COVID-19: Implications for human resource management	73	36
9	Tourism in a world with pandemics: local–global responsibility and action	70	35
10	Hospitality, tourism, human rights and the impact of COVID-19	70	35

Other impactful articles include “*Feverish stock price reactions to COVID-19*”—[21], which provides evidence of the impacts of COVID-19 on stock returns across US industries. The next is “*Effects of COVID-19 on hotel marketing and management: a perspective article*”—[54] which discusses the effect of COVID-19 on hotel marketing and management by outlining relevant research agenda to foster knowledge development. The multidisciplinary nature of COVID-19 research is evidenced in the diverse perspective from which these studies have examined the effect of the pandemic. Table 2 summarizes the top 10 most impactful studies.

The top 10 cited articles on COVID-19 and performance are presented in Table 8. The table indicates that the two most cited authors are Ivanov D and Wen J. who appear as the Top 2 and Top 18, respectively, in the authors with most publication list in Table 4. Most of the other authors in the most cited author list also appear in the most productive author list and these results signify that most of the authors in the two lists are not only productive but they are also very influential.

**Table 8:** Top 10 cited authors of Covid-19 and Performance

Author	No. of cited times
Ivanov D	448
Wen J	170
Jiang Y	92
Ratten V	64
Sharma A	43
Liu Y	35
Yang K	28
Zhang J	22
Aguinis H	19
Guthrie J	17

### Co-citation analysis

#### Articles

**Fig. 3:**
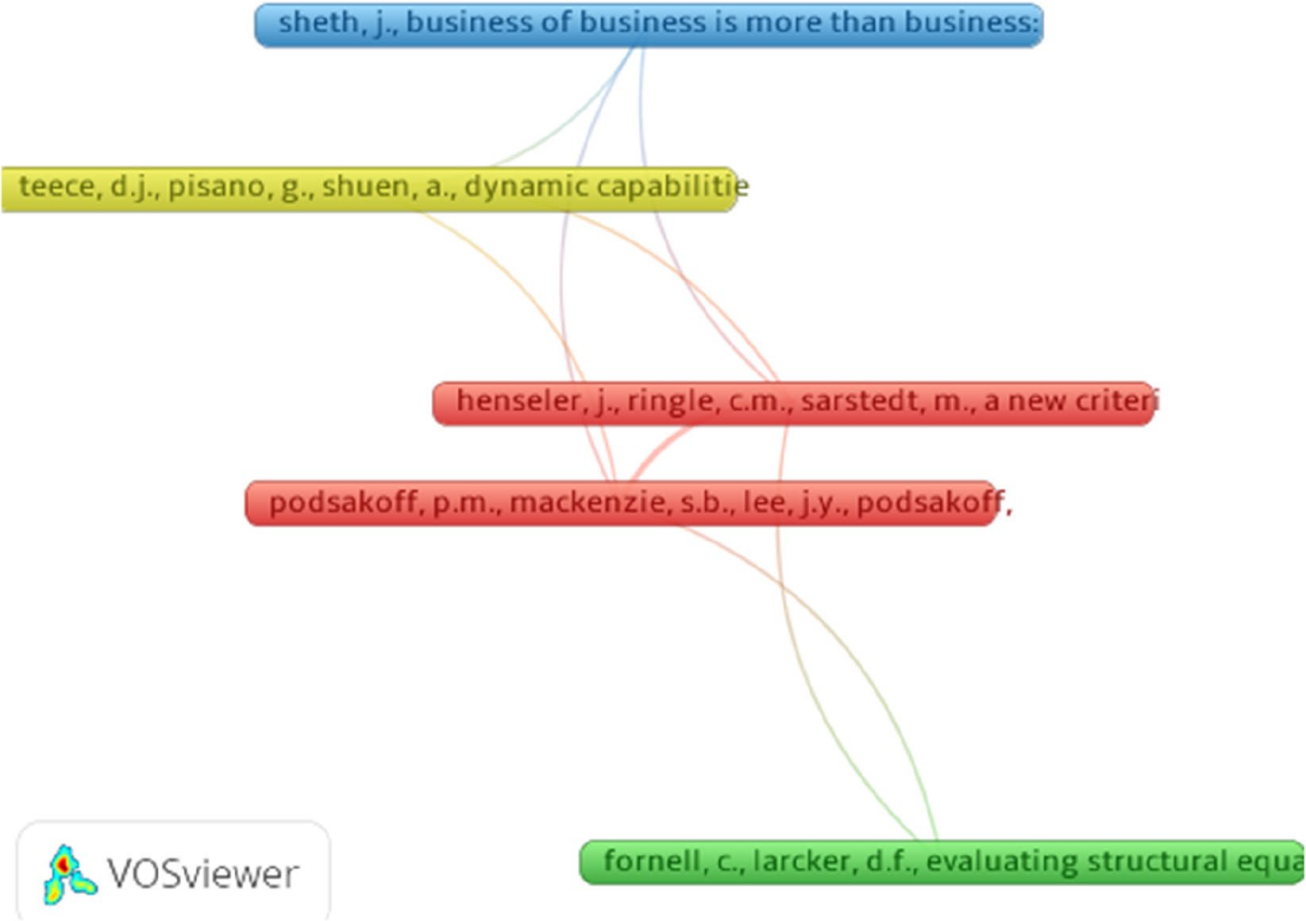
Network visualization of the largest connected sets of cited references

We analyze the 1355 articles in our dataset, with a minimum threshold of 3 citations; the obtained set contains 29 cited references out of the 6724 total references as reported by VOSviewer. The five most connected references (Fig. 3) are: Fornell, C. and Larcker, D.F., 1981. Evaluating structural equation models with unobservable variables and measurement error. *Journal of marketing research*, *18*(1), pp. 39–50. Henseler, J., Ringle, C.M. and Sarstedt, M., 2015. A new criterion for assessing discriminant validity in variance-based structural equation modeling. *Journal of the academy of marketing science*, *43*(1), pp. 115–135. Podsakoff, P.M., MacKenzie, S.B., Lee, J.Y. and Podsakoff, N.P., 2003. Common method biases in behavioral research: a critical review of the literature and recommended remedies. *Journal of applied psychology*, *88*(5), p. 879. Sheth, J., 2020. Business of business is more than business: Managing during the Covid crisis. *Industrial Marketing Management*, *88*, pp. 261–264. Teece, D.J., Pisano, G. and Shuen, A., 1997. Dynamic capabilities and strategic management. *Strategic management journal*, *18*(7), pp. 509–533.

Figure 3 displays the network visualization of the largest connected sets of cited references. The papers with the highest coupling strengths are those by Henseler, J., Ringle, C.M. and Sarstedt, M., 2015 and Podsakoff, P.M., MacKenzie, S.B., Lee, J.Y. and Podsakoff, N.P., 2003. These papers are central because of their specific contribution to methodological issues in business management and accounting research. Henseler et al. [55] provide guidelines on how to handle discriminant validity issues in variance-based structural equation modeling, while Podsakoff et al. [56] provide recommendations for how to select appropriate procedural and statistical remedies for different types of research settings.

#### Journal

**Fig. 4:**
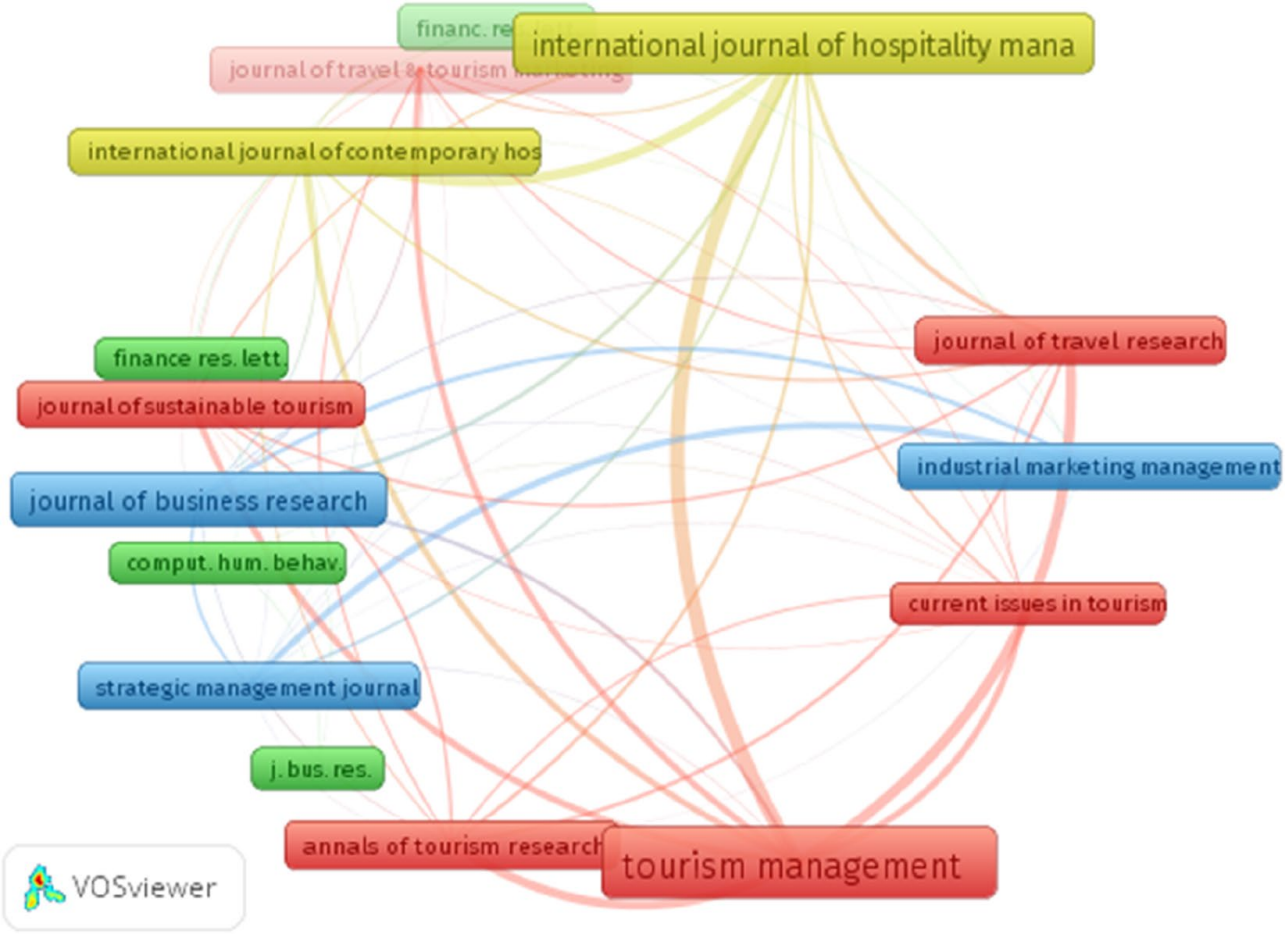
Network visualization of the largest connected sets of cited sources by journal

Out of the 2354 cited sources, 17 journal each received more than 20 citations. The top 5 journals with the highest numbers of citations are: Tourism Management (98), International Journal of Hospitality Management (66), Journal of Business Research (50), Annual of Research (38), and Journal of Travel Research (32) (Fig. 4). These numbers make it evident how much of the discussion of the impact of Covid-19 on performance is supported by papers published in Tourism Management. The analysis of the network visualization provides interesting considerations. It suggests the existence of four different clusters, regarding the managerial implications related to tourism, finance, marketing and hospitality management. The specific clusters on tourism and hospitality management is inevitable due to the impact of Covid-19 on this industry.

#### Authors

**Fig. 5:**
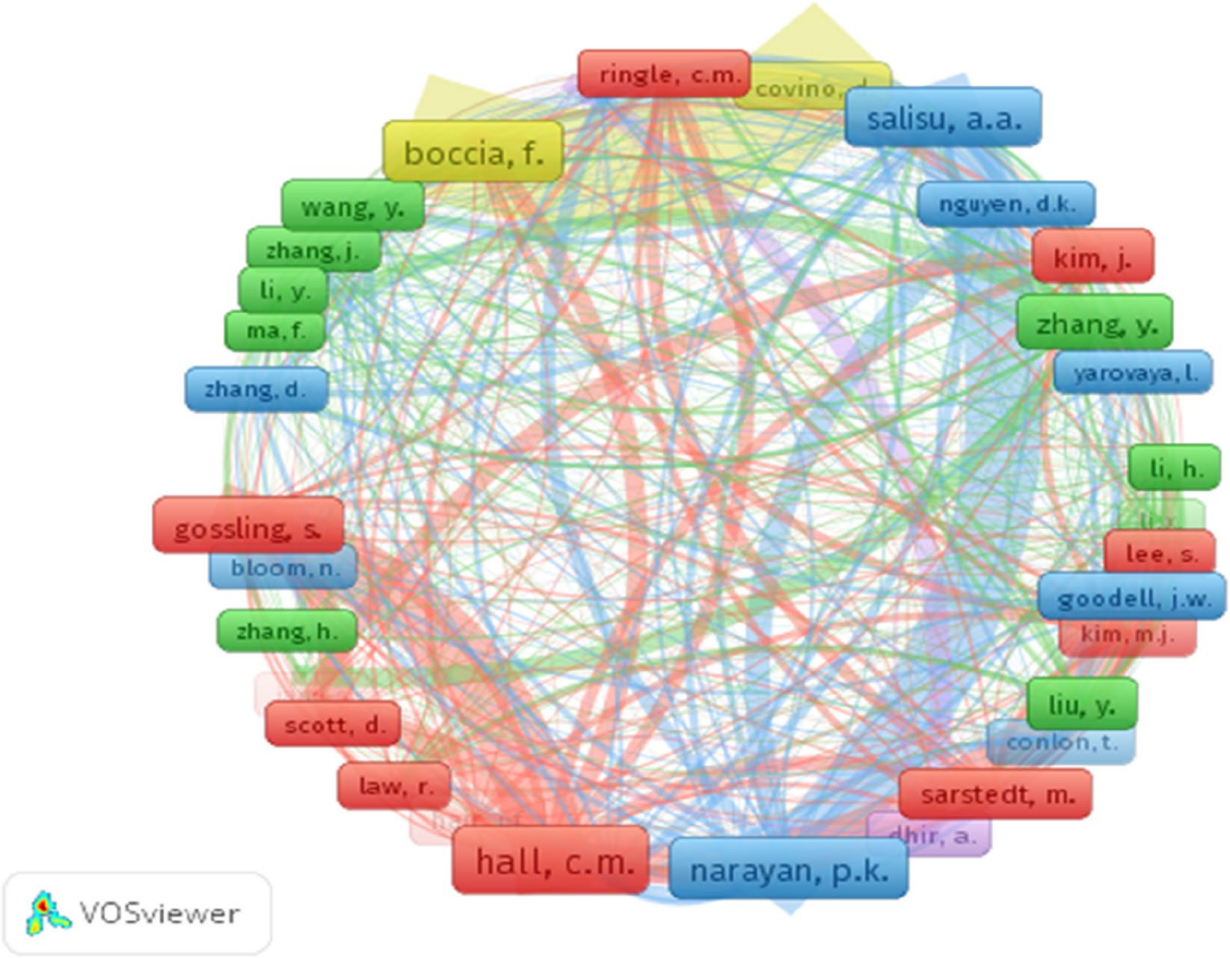
Network visualization of the largest connected sets of cited sources by authors

Out of the 9338 cited authors, only 52 had been cited more than 10 times, while only 9 authors were cited more than 20 times. The authors are: Hall C.M (38), Boccia, F (31), Narayan, P.K (31), Salisu, A.A (28), Glossling, S (25), Zhang y (25), Liu Y (22) and Sarstedt, M (20) from University of Canterbury, Parthenope University of Naples, Deakin University, University of Ibadan, Lund University, Harbin Institute of Technology, University of Illinois, Otto-von-Guericke University, respectively. Figure 5 shows network visualization of the author co-citation analysis. It also highlights that these authors are most connected as well as most cited. The network visualization displays the existence of five different clusters. The red, green, and blue clusters are characterized by a high degree of bibliographic coupling with 15–16 items each, while two clusters show 2 items each. The red, green, blue, yellow and violet clusters with the highest degree of bibliographic coupling contain Hall et al. [57], Zhang et al. [58], and Narayan et al. [59], respectively. These studies provide insights on the impacts of Covid-19 on the services sector and consumption displacement (Hall et al. 57), global financial markets (Zhang et al. 58) and economic stimulus (Narayan et al. 59) (Fig. 5).

**Fig. 6:**
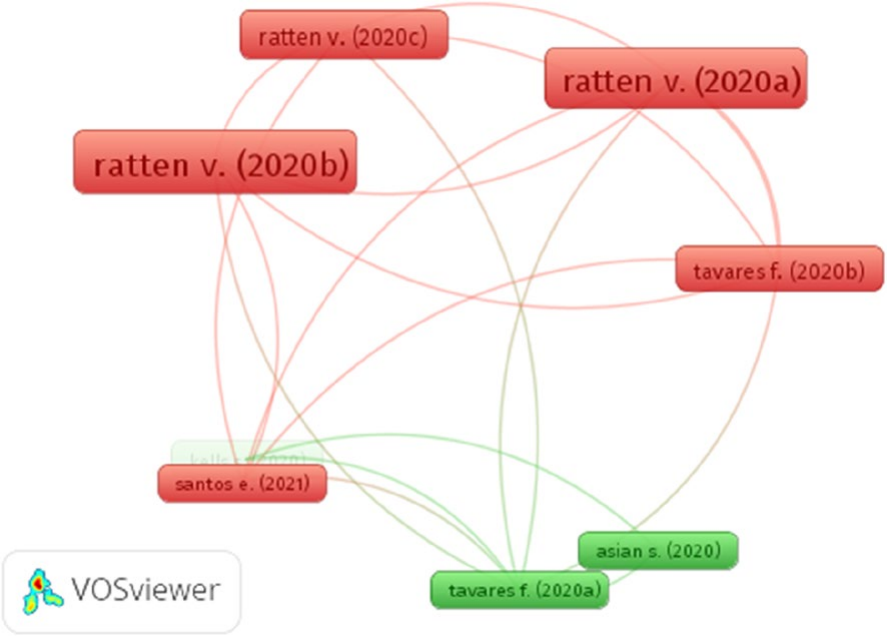
Network visualization of the bibliographic coupling of articles

### Bibliographic coupling

#### Articles

We use bibliographic coupling of the 1349 articles to understand the theoretical foundations of publications on Covid-19 and performance. The minimum number of two articles was analyzed, resulting in the most extensive set of connected documents of 418 publications (30.98% of the dataset). Figure 6 shows network visualization of the bibliographic coupling analysis by articles, highlighting two clusters with the largest set of connected articles of 8 publications (0.59% of the dataset) implying the absence of a consolidated Covid-19 on performance field of study. The red cluster is characterized by a high degree of bibliographic coupling with 5 publications (i.e., [60–63]) (Tavares et al. 64), while green cluster shows 3 publications (i.e., Asian et al. 65; Kells 66 ; Tavares et al. 67).

**Fig. 7:**
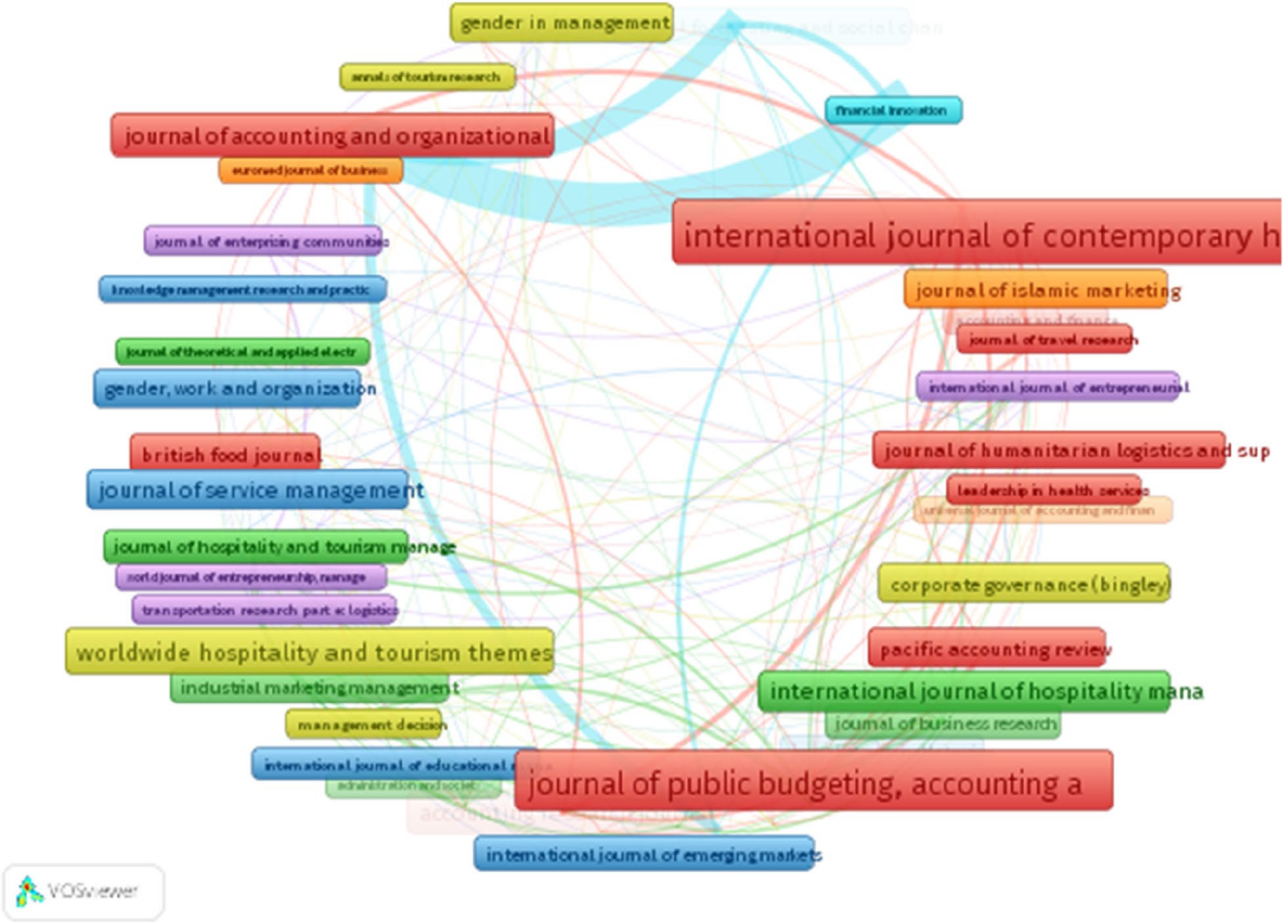
Network visualization of the bibliographic coupling of journals

#### Journals

To analyze the bibliographic coupling of journals, we set a minimum of two articles per journal (see [28]) (Ferreira 68), resulting in 226 (50.78% of the dataset) out of 445 journals (see Fig. 7). Figure 7 shows network visualization of the bibliographic coupling analysis by journals, highlighting seven clusters with the largest set of connected journals of 74, implying the absence of a consolidated Covid-19 on performance field of study. Figure 7 reveals that the five journals with the highest bibliographic coupling index are Research in International Business, Financial Innovation, Technological Forecasting and Social Change, Tourism Management, and Journal of Business Research. Figure 7 reveals the central role played by other journal in the various clusters including red (International Journal of Contemporary Hospitality Management and Journal of Public Budgeting, Accounting and Financial Management), violet (International Journal of Entrepreneurship Behavior and research), yellow (Corporate Governance-Bingley), green (Journal of Theoretical and Applied Electronic Research) and blue (International Journal of Emerging Markets). These show the field of the research on the impact of Covid 19 on financial and non-financial indicators is receiving attention from multi-disciplinary scholars of business management and accounting.

**Fig. 8:**
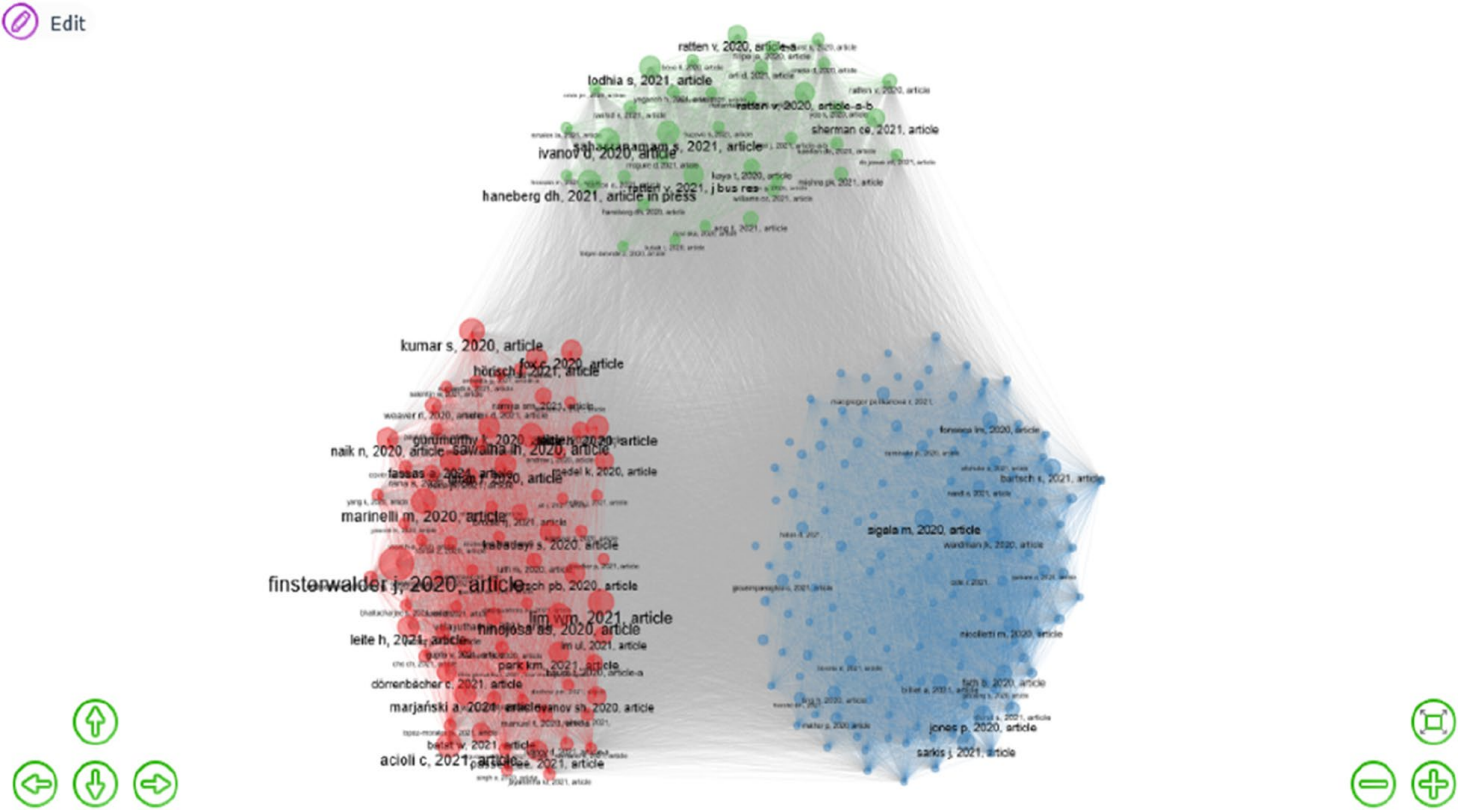
Cluster analysis of articles

## Thematic/cluster analysis

Next, to provide a deeper synthesis and identify relevant patterns in performance-related COVID-19 research, the study conducts a thematic analysis as advocated by [69]. The study begins by conducting a cluster analysis of the reviewed studies and subsequently discusses the themes of these clusters in detail. The study conducts a cluster analysis of articles to identify relevant ideas, couplings or themes shaping research in COVID-19 influence on performance research. Using a co-citation cluster analysis with Biblioshiny in R, the study identifies three main clusters of research. These are illustrated in Fig. 8.

Although identifying the main clusters of research in this domain is important, a deeper analysis of the identified themes or clusters will offer more critical insights to knowledge while guiding direction for future research. Thus, we discuss landmark publications within each thematic cluster and synthesize their key contributions to COVID-19 and performance literature. These publications are divided into three clusters contributing to different thematic areas within COVID-19 and performance literature, as identified in Fig. 3. The landmark publications highlighted within the first cluster assess the COVID-19 pandemic situation from various foundational perspectives. Cluster two contains articles focused on the broad concepts of crisis management and strategic management. Cluster three includes landmark publications which capture the performance outcomes and strategies of COVID-19 on various businesses including sports, education, and global supply chain.

### Cluster 1: foundational discussions and risk assessment

Cluster 1 is made up of several papers that assess the COVID-19 pandemic since its inception from various perspectives. These studies elaborate on the nature and implication of the pandemic on macro-economic management [70] hospitality and human rights to movement [71] as well as service ecosystems [44, 72]. Studies in this cluster provide an elaborate assessment of the COVID-19 pandemic, as a crisis with catastrophic implications while highlighting the shortcomings of current crisis management strategies worldwide on a business and macro-economic level.

First, we consider the seminal paper [71] which attempts to assess the impact of COVID-19 on human rights to participate in hospitality and tourism, as a result of imposed travel restrictions. The study assesses the extent to which government responses to the pandemic influenced individual right to travel for leisure, business, education among others. These were evident in the closing of tourist sites, national borders, recalling of citizens to their primary residence as well as national restrictions on movement, thus confining people to their homes with little mobility to almost all service provision locations except those considered essential. In certain instances, these restrictions resulted in the inability of some tourists to return to their home countries as was experienced on several cruise ships in Europe, the Americas and Asia. These restrictions additionally, resulted in the loss of employment of numerous individuals. However, the study shows that although the pandemic and its resultant restrictions have imposed numerous challenges to tourism, individual, business and national economic growth, the closure of national borders has seen a reduction in human trafficking, child sex tourism as well as the reduction in environmental pollution and degradation through fossil fuel consumption among others. Lastly, speculating that the global hospitality and tourism industry will face a precarious future riddled with mass closures of small hospitality businesses, increasing operating and consumer costs, the study urges that scholars continue to seek answers to important questions overtime on reinstating hospitality and tourism in a post-COVID-19 world.

Following the broad picture by Baum and Hai [71], Kabadayi et al. [72] synthesized the macro-economic impact of COVID-19 while offering a framework for recognizing impacts of disruptions on service ecosystems. The study proposes the concept of *service mega-disruptions (SMDs)* to refer to the simultaneous multi-industry service disruptions caused by a pandemic. Defining the concept as an event caused by an unforeseen pandemic which affects multiple stakeholders and service ecosystems simultaneously and remains difficult to swiftly recover from, the study introduces a multi-level framework which may better arm service researchers and practitioners alike for future similar disruptions. The study uncovers five research themes relevant in the reducing the impact of service mega-disruptions. These include service ecosystem recovery, service agility and transformation, service technology and automation, remote service provision and finally service theory of social distancing. The study provides these holistic themes which encompass the micro- (individual and employee), meso- (service industries and public services) and macro-level (government actions and policies) perspectives of recovery measures.

Next, Finsterwalder and Kuppelwieser [44] explore the impact of crises such as COVID-19, on the service industry and its research community. By identifying and categorizing the micro-, meso- and macro-levels of service ecosystems, the study introduces a novel resource-challenges equilibrium (RCE) framework for pre-incident, incident, and post-incident phase strategies directed at building resource resilience. The study highlights the need for stronger resilience to create, facilitate and leverage on safe co-creation spheres with consumers, businesses, not-for-profit organizations as well as governmental institutions. The study highlights the need for co-creation spheres, while accentuating the need for relevant resource-challenge balance to ensure business profitability as well all overall ecosystem equilibrium.

Next, the study discusses the article Andrew et al. [70], which explores the constraints of the Australian government in responding to crises with relevant budgetary action. The study reviews literature on the COVID-19 crisis, as well as public budgeting responses to the health and economic effects of the crisis. The study identifies public budgeting as being neoliberal. This has been evident in the duo phased response strategy to COVID-19 within the Coronavirus Economic Response Package Omnibus Bill (2020) by initially stimulating businesses through the instant asset write-off scheme in the phase first, and individuals through unemployment benefits among others in the second phase. The study in its examinations seeks to offer insights and synthesis of knowledge relevant to other countries in managing and mitigating the fiscal consequences of the COVID-19 crisis. By discussing responses, outcomes and shortcomings the study provides an overview of neoliberalism influenced crisis responses for the objective assessment of multiple governmental responses available.

Lastly, Ivanov and Dolgui [73] assess the state of global supply chain in the wake of the COVID-19 crisis and provide a methodical taxonomy of supply chain disruptions caused by pandemics. The study highlights the ideas of ripple effects, structural dynamics and network resilience relevant in the COVID-19 supply chain disruption discourse. The study assesses the various ripple effects caused by the pandemic, such as the halting of production by Chrysler Automobiles NV and Hyundai as a result of the lack of parts supplied from China. The study focuses on disruption propagation throughout networks also known as ripple effects and resultant changes within supply chain structures (structural dynamics) from an operational research perspective. The study reviews relevant theories and methodologies to disruption research at network, process and control levels. By reviewing resilience in supply chain literature, the study advocates for the consideration of 5 stages in building resilience. These include anticipation, early detection, containment, control and mitigation and finally elimination. The study thus, provides relevant direction for future research while providing foundational discourse to drive this increasingly important domain of supply chain studies.

Baudier et al*.* [74] use survey method to extensively examine the adoption of telemedicine solutions by patients in several countries in Europe and Asia to help avoid the spread of the disease and alleviate the associated impacts of the pandemic, while ensuring a relatively uninterrupted healthcare service provision. The study argues that the development of ICTs, the individual’s adoption rate of devices (tablets, computer, smartphones), the technological advancements of telemedicine tools, and, recently, the worldwide pandemic (COVID-19) are the key drivers of the expansion of healthcare services. The empirical results based on some constructs of the Technology Acceptance Model, Availability, Personal traits, and Perceived Risks emphasize the huge influence of Performance Expectancy, the positive impact of Contamination Avoidance and the negative effect of Perceived Risk on the adoption of Teleconsultation Solutions. Brodie et al. [75] similarly review the healthcare system, while highlighting the need for a sustained value co-creation perspective for healthcare delivery with the help of integrative technologies. This, the study argued, helps to create stronger resilience through knowledge sharing, flexibility information and learning. These studies highlight the integral need and potential ecosystem resilience has in responding to and mitigating adversity and crisis during a pandemic.

### Cluster 2: crisis and strategic management

The second cluster contains a number of articles which highlights the relevance of crisis and strategic management as well as communication, coordination and the media among firms. Kraus et al. [24] in a qualitative study of family firms in five western European countries, the study examined several strategic and crisis management measures used in adapting to the crisis. The study examined strategic crisis responses including retrenchment, persevering innovating and exit as discussed by Wenzel et al. [4]. Several firms included in the study begun changing or extending (innovating) their existing business models to take advantage of new consumer demands even though they may have lost a significant portion of their typical revenue streams. Others, however, continued to persevere by maintaining existing business models as a result of extensive investments in systems prior to the pandemic. The study highlighted various changes occurring among these firms. These include an increase solidarity and commitment among employees as well as a focus on increased digitalization. More prominently, although the study provided empirical evidence for and extended the strategic responses proffered by Wenzel et al. [4], results show that firms use a combination of various coping mechanisms for two main reasons: safeguarding liquidity and improving long-term survival and viability of the company. One strategic response is incapable of achieving both objectives, thus providing a basis for the combination of various strategic responses. The study however showed that, in the beginning stages of the pandemic no firm adopted exiting as a coping mechanism.

The tourism and hospitality industry has received the greatest brunt of the pandemic and as such continues to enjoy a burgeoning interest in performance, and management research. Although various studies have focused on the performance setbacks encountered by firms within this industry [76], Sigala [53] highlights the need to effective crisis management strategies. The study attempts to provide transformational remedies by unraveling all aspects of the industry including demand, supply, and other important stakeholders through three identified stages of responding, recovering and restarting. Giousmpasoglou et al. [77] extend this conversation by highlighting the relevance of managerial roles in effective crisis management. By ensuring that managers anticipate, equip and prepare their teams for crises by identifying, monitoring and mitigating potential vulnerabilities, firms within the hospitality industry will be better placed to manage crisis and reduce economic losses. By expanding this conversation to human resource management, Carnevale and Hatak [78] advocate for greater support to the workforce as they cope with altered work systems and environments and navigate changing work-family dynamics among others.

Again, we discuss studies in this cluster that highlight how countries and institutions deal with the impacts of the pandemic through communication and media. For example, Viola et al. [79] use survey data and the logit model to examine the effectiveness of institutional communication in mitigating COVID-19 impacts in Italy. The study also highlights the crucial roles of education, health literacy and the effect of asymmetric information on the effectiveness of institutional communication. The empirical results show that education plays a significant role in understanding communication pillars and building an individual consciousness about the pandemic and its associated impacts. Similarly, Machmud [80] by means of content analysis, assesses government officials’ communication and coordination intensity on twitter social media in dealing with the impacts of Covid-19 pandemic in Indonesia. The study documents that government officials are intensively building coordination and communication to overcome the performance impact of Covid-19 in Indonesia. The study further shows that the Indonesian President constantly communicates with the national COVID-19 team to ensure that all government agencies at both central and regional levels are actively mobilized and united. The study confirms coordination and communication strategic crisis management vehicles that enable public officials to jointly implement COVID-19 control policies quickly and accurately throughout Indonesia.

We also highlight studies that contribute to the theoretical development on the use of media by individuals to deal with the impacts of the pandemic. By adopting the theory of planned behavior (TPB), Mohammed and Ferraris [81] analyze the role of social media in reducing the effects of the pandemic by specifically looking at the factors that stimulate individual’s participation in social media during crises. The empirical method from the survey data shows that attitude, perceived behavioral control, subjective norm, hedonic, utilitarian values and trust affect Twitter users’ active participation significantly during the pandemic. The understanding of these driving factors could help enhance user participation, and information dissemination in the era of social distancing and lock-downs. In the same vein, Kim [82] employs survey data to examine the effect of video games on the psychological of individuals in the era of COVID-19. The study finds that video individual’s negative and positive emotional states while playing a video game increase one’s level of psychological well-being, which also results in loyalty toward the video game. The empirical findings also indicate that individuals’ positive and negative emotional states while playing a video game were obtained from the perceived emotional value of the virtual product, implying that people evaluate the game not only based on time, money and effort, but also based on enjoyment, positive feelings and pleasure from consuming the digital product. The psychological benefits derived will improve the level of positive emotions and reduce the levels of negative emotions while partaking in the recreational activity. The study advocates that video game companies should design more exciting games that offer fun and entertainment to consumers to help improve the psychological well-being of consumers during this crisis.

The last strand of studies in this cluster emphasizes the role of media in overcoming the performance impact of COVID-19 across industries. These studies highlight the significance of the media in enhancing performance of retail supply chains, tourism, and brand engagement (e.g., [83–85]. For example, Im et al. [84] develop two joint models with fixed-effects estimations to examine the relationships among the pandemic, online information search, social distancing, and firm performance in the tourism and hospitality industries. The first model explored the relationship among COVID-19, information search, social distancing and stock performance of tourism and hospitality companies. The results reveal that news coverage on COVID-19 significantly impacts information search and social distancing, and social distancing, in turn, exerts an impact on stock performance. The second analysis focused on the effect of the pandemic on hotel reviews through information search and social distancing for tourist attractions at the regional level. The results indicate that when looking at the geographical effect, news coverage and the number of confirmed cases both lead to variations in social distancing and information search for tourist attractions and these behavioral tendencies are influential in hotel selection. Thus, media coverage and the number of confirmed cases in the news significantly influence social distancing actions of consumers which in turn influences the stock performance of tourism and hospitality industries.

### Cluster 3: performance outcomes and strategies

The final cluster contains a number of articles which discuss recorded or projected effects of COVID-19 on various business setups while offering specific remedies, and perspectives for sustained resilience and stronger performance. In Ivanov [51] the impact of COVID-19 on global supply chains is examined using a simulation-based methodology to predict and examine disruption effects on supply chain performance. This groundbreaking study sets the basis for later empirical studies on supply chains resilience and performance in pandemic era. The study primarily analyzes the manner in which simulation-based methodology can be adopted to examine and predict the effect of pandemic on global supply chain performance. The study highlights the need for firms to be resilient against the disruptions, risks and uncertainties caused by the COVID-19 as such epidemics start small but scale fast and spread across vast geographical expanses creating uncertainty and resulting in numerous unknown and usually adverse outcomes. The results of the simulation based on primary and secondary data offers possibility of predicting both long-term and short-term supply chains performance impact of pandemic in different scenarios. The study approach helps to identify the successful and wrong elements of risk preparedness/mitigation and recovery policies when pandemics erupt. The study results indicate that the timing of the opening and closing of facilities at different strata may become a key factor that determines the impact of epidemic outbreak on supply chain performance rather than the speed of epidemic spread or the duration of an upstream disruption. That is, in the event of pandemic propagation, supply chain performance and reaction depend largely on the timing and the scale of disruption propagation and the sequence of facility opening and closing at various supply chains strata.

Ratten [60] reviews literature on crisis and its effects on entrepreneurship. By focusing more intently on cultural, lifestyle and social changes experienced in society, the study examines how entrepreneurship has changed in the wake of COVID-19. The study highlights the need for stakeholders to be proactive during a crisis, as it presents both an opportunity and a threat. The study advocates for entrepreneurs to build a social movement by considering broader community needs in addition to their business needs. Additionally, the study highlights the need for a focus on societal trends as well as social changes as a way to surmount possible business setbacks as a result of COVID-19, while taking full advantage of new opportunities. By focusing on inter-organizational networks and collaboration, the study posits that entrepreneurs will begin to leverage and create new, relevant and sustainable innovations. Through appropriate information and resource sharing policy can ensure that entrepreneurs are equipped with relevant tools to rejuvenate troubled industries and grow related businesses.

Ratten [62] examines the extent to which COVID-19 has influenced sports entrepreneurship, creating the need for considering new business models and encouraging creativity. The study examines the intersection between crisis management and sport entrepreneurship and provides concrete paths to resilience, growth and performance success in dealing with COVID-19. The study examines the concept of sports entrepreneurship, and highlights the critical role technological innovation has played in the success of businesses in this domain. Although the health crisis caused by COVID-19 has resulted in the postponement and cancelation of various sports games including Euro 2020, and the Olympics among others, the study suggests that firms may rise above these setbacks with investments in capital and infrastructure to encourage greater customer and fan engagement as a way to be more entrepreneurially oriented.

Ratten [61] considers the extent to which COVID-19 has influenced educational entrepreneurship in its almost complete shift to online learning. The global education system was profoundly affected in areas of service, research and teaching. However, the study proffers that educational innovation and the leveraging of multifaceted and rich digital learning environments provides sustainable means through which education communities may cope with the devastating effects of COVID-19. By reimagining online teaching and learning experiences, possibly including artificial intelligence-related tools, and adopting a complimentary approach of innovation and empathy, education communities will begin to identify new revenue streams while strengthening existing ones.

Next, we discuss seminal studies that focus on how entities are responding to the impacts of the pandemic for survival and sustainability (e.g., [63, 86–88]). Santos et al. [63] undertake a comparative study across nine countries to unravel the factors that affect COVID-19 infections and deaths across countries to sustain economies. The study specifically looks at socio-economic indicators and COVOD-19 testing, comparison of infection and death rates across countries and the impact of climate on infection rates across countries. The study finds a significant impact of climate change on COVID-19 infection rate. The results also indicate that socio-economic indicators such as security index, innovation, and GDP per capita are important for a country's sustainability, being imperative to respond to anxious moments such as what nations are living from the COVID-19 pandemic. Through content analysis, Hossain [89] investigates how the sharing economy (SE) is coping with the changing environment triggered by the Covid-19. The study examines SE sector from four main perspectives: service providers, SE firms, regulatory bodies, and service receivers (customers). The study also explores SE along the following themes: income reduction, anxiety, job loss, hygiene and safety, cancelation, overcoming strategy, and outcomes. The study results indicate the devastating impact of the pandemic on the performance of SE firms and service providers such as Airbnb, accommodation hosts, Uber, and their Uber drivers. Therefore, firms and service providers have adopted strategies to survive in business. The study indicates that because of the pandemic, accommodation hosts are looking at long-term tenants and focusing on domestic instead of foreign guests. This is mirrored by Airbnb strategy of beginning to focus more on long-term stays. These overcoming strategies significantly reduce the impact of the pandemic on SE performance.

## Directions for future research

Following the critical review of several relevant studies, this study seeks to categorize and highlight important gaps in literature as well as pertinent trends and foci which research may benefit from while offering practical knowledge and solutions for policy and practice. By structuring relevant gaps and research trends into unique categories, the study provides a means to decompose the broad research domain into vital and unique sub-domains, each warranting extensive and in-depth consideration. For instance, the cluster analysis reveals unexplored areas such as the use of digital technologies and big data to boost performance in the era of viral pandemic. For methodological gaps, the studies analyzed are limited in terms of collecting data at the early stages of the pandemic, hence, the need to consider longitudinal data to better understand the performance impact of pandemic. Another methodological gap is that most of the studies use case-study approach. For contextual gap, the cluster analysis shows lack of attention to supply chains reactions under different pandemic plans. The supply chain performance studies also omitted elements such as back-up suppliers, reserved capacities, regional sub-contracting and lead-time reservations, which could obscure managerial insight. The studies are also limited to upstream disruptions, which call for examination of pandemic disruption in downstream supply chains strata and the antecedent impact on forward and backward propagations of ripple effect.

To facilitate research in addressing the theoretical, contextual, and methodological gaps such as those highlighted, we implement a four-step approach to discern future research agenda by adopting content and bibliometric analyses (Bahoo, 89). First, we reviewed 50 top-cited articles that make a citation map. Second, we reviewed all the influential and trending articles during the last 6 months (January to July 2021). Third, we reviewed the remaining articles in our sample to circumvent top citation bias. Fourth, we transformed the possible research agenda into research questions and excluded those questions already investigated by researchers. This systematic process produced the 20 future research questions listed in Table 9. Through an in-depth qualitative and quantitative review, we recommend a need to establish an appropriate pandemic response framework to help businesses, governments and policy makers to maintain resilience besides maintaining public health safety during such crises.

**Table 9:** Future research agenda

SN	References	Research questions
1	Ivanov [51]	RQ1: How can we make innovations and data work for Supply Chain resilience in crisis times? RQ2: How can new digital technologies improve the ripple effect control in cases of epidemic outbreaks?
2	Sigala [53]	RQ3: How do crises accelerate technology innovation and change? RQ4: What is the impact of COVID-19 across all the actors of the same tourism stakeholder group? RQ5: What is the impact of COVID-19 on different market segments and stakeholders? RQ6: What are the effects of COVID-19 on employee psychological, mental and physical health, engagement, working conditions and other human resource issues within the tourism industry?
3	Ramelli and Wagner [21]	RQ7: What is the effect of the ongoing policy interventions and individual behavior changes on the economic recovery of countries worldwide?
4	Jiang and Wen [54]	RQ8: What are the effects of big data and analytics (i.e., AI, hygiene and healthcare practices) on digital transformation in the hospitality industry? RQ9: Do technology-based approaches affect service disruptions and failure and guest loyalty, in the post-Covid era? RQ10: What are the different approaches via which hotels could work with governmental agencies to develop coordination mechanisms and comprehensive crisis management schemes?
5	Kraus et al. [24]	RQ11: To what extent is an initial discussion of a business model innovation during a crisis affect actual implementation in the long run? RQ12: How are family managers assessing the current crisis situation, and how decision-making about coping mechanisms is utilized in various family situations?
6	Carnevale and Hatak [78]	RQ13: Does COVID-19 affect employee adjustment and well-being?
7	Baum and Hai [71]	RQ14: Does Covid-19 affect human rights in the context of hospitality and tourism?
8	Jamal and Budke [90]	RQ15: What are the effects of the 2019-novel coronavirus on Countries and destinations using both monetary and non-monetary metrics?
9	Lai and Wong [91]	RQ16: How does epidemic crisis strategies for boutique hotels and resorts differ across hotel segments?
10	Jones and Comfort [92]	RQ17: How and where can we integrate local aspirations and values into corporate approaches to sustainability, in the aftermath of the COVID-19 crisis, using case studies of local hospitality managers within less developed counties?
11	Kabadayi et al. 72	RQ18: To explore how and when service companies adopt technology and automation in response to SMDs such as COVID-19?
12	Zhang et al. [58]	RQ19: Where are the antecedents of employee safety behavior from organizational and individual levels in the era of Covid-19?
13	Ahrens and Ferry [93]	RQ20: What are the tactics used as the crisis unfolded by both local and central governments across the globe?

The table shows 20 future research questions. RQ denotes research question

## Conclusion and limitations

The study provided an integrative review to map out the COVID-19 performance discourse. The study adopts a systematic approach to identifying relevant literature used in this review. The study performs a performance analysis to identify seminal studies, impactful authors and high-ranking journals and affiliations. Additionally, the study provides a geographical science mapping of research attention. To guide future research direction, the study conducts a keyword analysis and cluster analysis to identify relevant research themes.

Although the study makes relevant contribution to knowledge, and highlights impactful scholars in the field, the list of impactful scholars provided in this article is far from exhaustive. Additionally, the study adopts a combination of the H, G and M indexes as these indices provide some idea of the impact of authors, it must be noted that such analyses are not without fault. As such, future studies may adopt a wider combination of robust indices in assessing performance factors of authors, articles and journals. The research on the impact of covid-19 on firm performance is now developing so the review in this study focused on all firms. Thus, future reviews may look at how the pandemic affects performance of specific firms and/or sectors since different firms may have different growth objectives, which could influence the impact of COVID-19 on these firms.

## Data Availability

The data that support the findings of this study are available on request from the corresponding author.

## Abbreviations

COVID: Coronavirus disease
USA: United States of America
UK: United Kingdom
SMDs: Service mega-disruptions
RCE: Resource-challenges equilibrium
ICT: Information and communication technology
SE: Sharing economy
GDP: Gross domestic product
